# Suitable indicators using stem diameter variation-derived indices to monitor the water status of greenhouse tomato plants

**DOI:** 10.1371/journal.pone.0171423

**Published:** 2017-02-03

**Authors:** Zhaojiang Meng, Aiwang Duan, Deli Chen, Kithsiri Bandara Dassanayake, Xiaosen Wang, Zugui Liu, Hao Liu, Shengguo Gao

**Affiliations:** 1 Farmland Irrigation Research Institute, Chinese Academy of Agricultural Sciences, Xinxiang City, Henan, P.R China; 2 Melbourne School of Land and Environment at the University of Melbourne, Melbourne, Australia; University of Delhi, INDIA

## Abstract

It is very important to seek a simple nondestructive method to continuously measure plant water status for irrigation scheduling. Changes in stem diameter in response to plant water status and soil water content (SWC) were experimentally investigated during the growing seasons of 2011/2012 and 2012/2013 in pot-cultivated tomato (*Lycopersicon esculentum* L.) plants in a plastic greenhouse. This study was conducted to determine suitable SDV (stem diameter variation)-derived indices as indicators of tomato plant water status for irrigation scheduling. The experiment was designed as a two-factor randomized block using the SWC and growth stages as variables. The SWC was controlled at 70–80% (well-watered), 60–70% (slightly deficit watered), 50–60% (moderately deficit watered) of the field capacity (FC), and the prescribed growing stages were vegetative, flowering and fruit-forming, and harvesting stages. Regression analysis showed that the SD_6_ (the difference between the stem diameter value at 06:00 am and the initial sensor reading) was closely related to the SWC (p<0.01) during rapid vegetative growth, whereas the MDS (the maximum daily shrinkage) was closely related to the SWC (p<0.01) during slow vegetative growth. Our results suggest that SDV-derived indicators can be used for determining plant water status and for scheduling irrigation at different growth/developmental stages.

## Introduction

The monitoring of plant water status provides an important source of information for irrigation scheduling. Therefore, a simple, stable, nondestructive method of continuous monitoring of plant water status has long been sought in research on soil-water-plant relations. Such a method is needed for studying the influence of various environmental factors on water status and subsequent plant growth. Numerous methods of measuring plant water status are recognized among scholars and experts. However, there has been no general agreement on the most suitable indicator [1]. Pre-dawn leaf water potential [2, 3] is frequently used, while other indicators such as stem water potential [4, 5], vapor diffusion methods and relative leaf water content have been adopted. Most of these methods require destruction of plant tissue and all provide intermittent and localized measurements rather than continuous and nondestructive monitoring of plant water status, which may have curtailed the adoption of these techniques for the calculation of irrigation requirements for large areas of farmland.

A reliable index of plant water status can only be obtained from plant measurements [6]. Since the plant water status controls many physiological processes and crop productivity, this information can be highly useful in irrigation scheduling. The continuous control of plant water status is crucial, particularly under deficit irrigation conditions, to prevent a moderate and potentially beneficial water stress from becoming too severe and reducing yield. For these reasons, monitoring the response of the entire plant to water status based on stem diameter variation has become popular in the field of irrigation management worldwide [7–17].

Many researchers around the world are devoted to identify useful indicators based on changes in stem diameter and its threshold values in response to plant water status for irrigation scheduling. Ortuno et al. [18] showed that when trunk growth was very low, the maximum daily trunk shrinkage (MDS) was the best indicator, while the minimum trunk diameter (MNTD) and maximum trunk diameter (MXTD) were the most reliable indicators under more rapid growth conditions of citrus trees. Nortes et al. [19] indicated that the MDS and trunk growth rate (TGR) were sensitive to water stress and that the TGR was useful as an indicator of stress and could serve in aiding irrigation decisions for young almond trees. The results of an experiment conducted by Moreno et al. [20] on adult olive trees showed that it is possible to obtain baseline values for the MDS, and the MDS behavior was best correlated with midday vapor pressure deficit and midday air temperature. The MDS signal intensity (actual MDS/reference MDS) threshold values are suitable for adjusting an irrigation schedule based on work conducted on almond [21, 22], lemon [23–25], adult Fino lemon [26], citrus [27] and lemon trees [7]. Swaef et al. [8] determined reference values for stem water potential and maximum daily trunk shrinkage in young apple trees based on plant responses to water deficit. According to Cuevas et al. [9], the MDS and DR (daily recovery) signal intensity may be useful indicators for the avoidance of fruit shriveling in deficit irrigated olive orchards for the production of good quality oil. In addition, reliable reference equations for irrigation scheduling using the signal intensity approach were obtained from the regression of MDS values vs. the daily maximum air temperature and the vapor pressure deficit of the air. Moriana et al. [10] reported that midday stem water potential (SWP) was a superior plant-based water status indicator compared with the TDF (trunk diameter fluctuation) parameters when deficit irrigation scheduling was not performed, and the difference in the TGR (DTGR) appeared to be a good indicator of water stress based on a threshold value of approximately −1.4 MPa in olive trees. Badal et al. [11] proposed that the MDS was a sensitive indicator of Kaki tree water status and could be further used as an irrigation scheduling indicator for optimum irrigation management of this crop; however, large MDS tree-to-tree variability should be taken into account when selecting the number of trees to monitor within an orchard. Livellara et al. [12] reported that the threshold value of the water deficit was associated with a critical stem water potential (SWP) value of −0.50 MPa; the use of this critical SWP value and its correlation with the MDS and TGR resulted in critical values of 165 μm and 86 μmm day^−1^ for MDS and TGR, respectively.

However, most recent work on SDV-derived indices as indicators of plant water stress was conducted on horticultural tree species. In contrast with the large number of studies in fruit trees, only a few studies have examined herbaceous species [28–31], whereas very few studies have assessed the SDV on vegetables such as tomato [32, 33]. Consequently, the main objectives of this work were (i) to characterize the behavior of the stem diameter variation and its relationship with other plant-based water indicators at different phenological stages in tomato, (ii) to determine SDV-derived indices as indicators and threshold values of plant water status in tomato for irrigation scheduling, and (iii) to analyze the relationships between stem diameter variation and meteorological variables during slow vegetative growth of tomato plants to establish a simulation model based on multiple factors for quantitative monitoring of the plant water status. This will provide a theoretical basis and technical parameters for further study and to determine useful indicators based on stem diameter variation in response to plant water status for automatic irrigation of different plant species under various ecological conditions.

## Materials and methods

### Experimental site and cropping details

The tomato pot experiment was conducted during the growing seasons of 2011/2012 and 2012/2013 in a plastic greenhouse (40 m long by 8.5 m wide) at the Experimental Farm in Crop Irrigation of the Farmland Irrigation Research Institute of the Chinese Academy of Agricultural Sciences (35°19^′^ N, 113°53^′^ E, 73.2 m elevation), in Xinxiang City, Henan Province, China, in the Huang-Huai-Hai Plain. The climate is typical temperate, and the area is semi-arid to semi-humid. The mean annual air temperature is 13.5°C, the annual accumulated temperature above 0°C is 5070.2°C, annual sunshine duration is 2497 h, the frost-free period is 220 days, precipitation is 580 mm, and potential evaporation (measured with 20-cm pan) is 2000 mm, based on 50-year weather data averages collected at the Xinxiang Weather Station in close proximity to the experimental site. The groundwater table is higher than 8 m. The soil is sandy loam with a mean bulk density of 1.38 g cm^-3^, a mean field capacity of 24% (gravitational content) and a mean permanent wilting point of 8% (gravitational content) in the 0–100 cm profile. In the greenhouse, the contents of soil organic matter, total N and P and available N, P and K were 18.85 g kg^-1^, 1.10 and 2.22 g kg^-1^ and 15.61, 72.0 and 101 mg kg^-1^, respectively. The unheated plastic greenhouse had an east-west orientation and was passively ventilated.

Tomato plants (*Licopersicum esculetum* L., Jindin No. 1, a local cultivar) were selected as the experimental material. Tomato seeds were planted on December 15 and the experiment was initiated using 10-week-old transplants. The cylindrical iron pots used in the experiment consisted of inner pots (29.5 cm diameter, 38 cm deep) and outer pots (31.0 cm diameter, 38 cm deep). The outer pots were embedded at a depth of 33 cm below ground (i.e., the rim of the pot was positioned 5 cm above the ground), and inner pots were placed in outer pots, which was convenient for weighing the inner pots. A layer of sand (5 cm thick) was placed at the bottom of the inner pot and used as a filter bed to adjust soil water and air conditions. Two perforated plastic tubes (3 cm internal diameter) were wrapped in gauze and placed in the sides of each pot for water supply or drainage. The soil in the inner pots was packed lightly to a bulk density of 1.25 g cm^−3^. The packed soil had a field capacity (FC) of 24% (expressed on a mass basis), an organic matter content of 9.30 g kg^−1^, a total N content of 0.98 g kg^−1^, and soil available N, P and K contents of 44.02, 6.2 and 112 mg kg^-1^, respectively. A compound fertilizer (20 g; N: P: K = 15:15:15) was added to each pot as a basal fertilizer to ensure sufficient nutrient supply during the experimental period. Tomato plants were transplanted into inner pots (one plant/pot) filled with loam soil.

### Irrigation treatments and experimental design

A two-factor randomized block design was used for the pots. The water treatment factor contained three levels of relative soil water content (SWC): 70–80% FC (Field water capacity), 60–70% FC and 50–60% FC, representing well-watered, slightly deficit watered and moderately deficit watered, respectively. The second factor was the various phenological stages of the plant, i.e., the seedling, flower-fruiting and harvest stages. There were a total of 9 treatments replicated 3 times, yielding a total of 27 pots. The pots were weighed daily using an electronic scale. All treatments received irrigation water according to designated levels (i.e., when the relative soil water content dropped below the designated lower limit, it was replenished to the designated upper limit) for each phenological stage, whereas all plants were well irrigated during other stages. The total volume of water applied during the experimental cycle was measured using a measuring cylinder. Evapotranspiration was determined using the water balance method.

In order to investigate dynamics of stem diameter variation in tomato and its relation to soil water content during drying cycle (from FC to wilting point), a drying pot experiment was also carried out simultaneously. This experiment consisted of 8 pots; 4 pots were used to attach stem diameter sensors for measuring stem diameter variation (SDV) and rest 4 pots were used for monitoring changes in soil water content by weighing method. The top soil surface in pots was covered with plastic sheet to curb evaporation. Prior to commencing drying cycle, pots were irrigated fully in the early evening, and the relative soil water content (SWC) was measured next day morning (through oven drying-method). Later set of 4 pots were weighed every morning and evening to determine water loss each day. Daily SWC was calculated from average values of morning and evening. When predawn leaf water potential decreased dramatically and reached wilting point, drying cycle was stopped and pots were well irrigated with a volume of water equivalent to 100% FC. A few days after the recovery, drying cycle of plants was again commenced.

### Measurements and methods

Daily meteorological data, including air temperature (T_a_), relative humidity (RH), and solar radiation (Rs) were recorded by an automatic weather station located in the greenhouse, and atmospheric vapor pressure deficit (VPD) was calculated from the T_a_ and RH data (Table 1).

**Table 1:** Daily mean values of main meteorological factors including air temperature (*T*_*a*_), relative humidity (*RH*), solar radiation (*Rs*) and vapor pressure deficit (*VPD*) during the period from April 8 to 15, and from June 8 to 15, 2012/2013 in greenhouse.

Month/Date	2012				2013			
	*T*_*a*_(℃)	*RH*(%)	*Rs*(MJ.m^-2^.d^-1^)	VPD(KP_a_)	*T*_*a*_(℃)	*RH*(%)	*Rs*(MJ.m^-2^.d^-1^)	VPD(KP_a_)
Apr								
8	18.62	54.54	5.42	0.84	19.83	89.95	1.04	0.20
9	20.10	60.00	4.81	0.80	25.04	77.87	13.92	0.58
10	18.06	69.02	3.35	0.55	22.32	77.10	10.90	0.52
11	19.67	72.88	3.23	0.53	19.58	82.86	5.58	0.33
12	20.11	75.62	3.93	0.49	17.86	86.85	5.09	0.23
13	18.97	75.93	3.09	0.45	22.58	76.95	12.42	0.53
14	20.85	66.73	4.61	0.69	20.89	80.18	7.37	0.41
15	16.27	75.00	4.46	0.40	23.16	73.84	13.18	0.62
Jun								
8	31.36	40.77	4.00	2.31	28.69	58.82	12.10	1.30
9	31.42	42.14	3.86	2.23	29.01	59.36	13.98	1.30
10	25.76	51.75	1.93	1.34	28.62	54.19	11.05	1.44
11	27.39	51.79	4.93	1.53	28.00	60.66	12.27	1.20
12	29.53	51.42	4.73	1.73	28.15	57.31	10.93	1.31
13	26.10	68.32	3.20	0.97	27.80	69.32	6.40	0.92
14	26.56	58.13	4.92	1.25	26.44	67.71	8.33	0.91
15	28.42	47.75	6.20	1.80	24.69	75.16	7.20	0.63

The SDV was continuously measured in three plants per treatment in different replicate pots throughout the experimental period using a set of linear, variable displacement transducers (LVDT) (model DF *±* 2.5 mm, accuracy *±* 10 μm, Solartron Metrology, Bognor Regis, UK) mounted on holders made of aluminum and ‘invar’ (an alloy of iron and nickel) with minimal thermal expansion. Sensors were attached to the main stem of selected tomato plants, approximately 10–15 cm above the ground level. The contact point of the sensor made contact with the surface of the stem, for which a spring and cyanoacrylate glue were used. The sensors were connected to a data logger (model CR10 X with AM416 multiplexer, Campbell Scientific, Logan, UT, USA) programmed to automatically scan the sensor outputs every 10 seconds and store average values every 30 minutes. The data logger was provided with a system for data transmission to the computer. Throughout the experimental period, sensors were reset at 3- (during active growth stages) or 5- (during non-active growth stages) day intervals.

The following indices were derived from continuous measurements of stem diameter variation: the maximum daily stem diameter (MXSD), minimum daily stem diameter (MNSD), maximum daily stem shrinkage (MDS) (i.e., the difference between the MXSD and MNSD) [21], and the daily variation in stem diameter at 06:00 am (SD_6_) (the difference between the stem diameter value measured at 06:00 am). Daily stem diameter growth rates (SGR) were calculated using MXSD values measured on two consecutive days.

The pots were weighed daily using an electronic scale for the control of soil water required by the treatments. Soil water content was determined gravimetrically. The leaf water potential (ψ_L_) was measured periodically (approximately hourly) on recent fully expanded leaves from the east and west sides of plants on which the LVDT sensors were placed; the average value of two samples was reported. The ψ_L_ was measured at mid-morning using a pressure chamber (model ZLZ-4). The leaf relative water content (the ratio between the actual water content and the one of turgid leaves as determined after rehydration, LRWC) was measured by oven drying and weighing. All plant measurements were made at the same time under clear conditions (08:00 to 18:00 h).

### Statistical analysis

All data are presented as means. Statistical analysis was conducted using Excel 2007 and DPS v 7.05. The differences between the treatments were examined using an ANOVA test. If statistically significant differences (P < 0.05) were detected using the ANOVA, a multiple comparison between means was performed using the Duncan method. Regression analysis was performed to establish a multiple regression equation between the MDS and environmental variables, and correlation analysis was used to determine the correlations between the SDV-derived indices, SWC, meteorological factors and other plant-based water status indicators. Differences were considered significant when P < 0.05 (represented as * in tables and figures) and extremely significant when P < 0.01 (represented as ** in tables and figures). Excel 2007 was used to create the artwork and Photoshop CS3 version 10.1 was used for further compilation.

## Results

### Dynamics of stem diameter variation in one drying cycle

**Fig 1:**
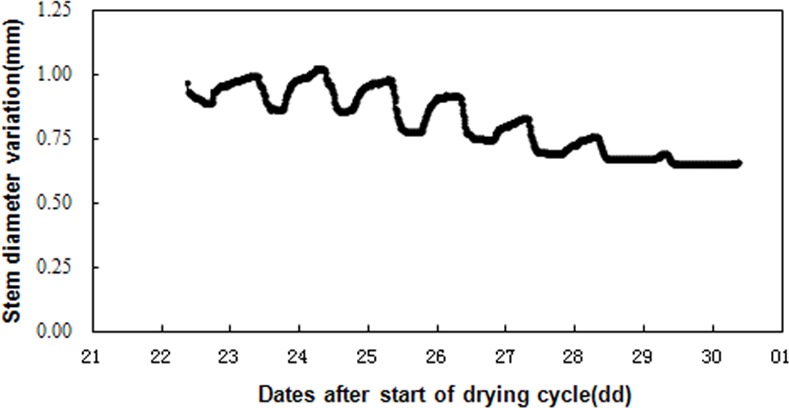
Dynamics of stem diameter variation (SDV) in tomato during one drying cycles (from FC to wilting point). Data were collected during a drought experiment conducted from June 22 to 30.

Fig 1 shows the dynamic of stem diameter variation (SDV) in drying cycle. Stem diameter in tomato plant showed a typical 24h cycle variation with ‘dentate shape’ fluctuation during a drying cycle (06-22-2011to 06-30-2011). On all sunny days the stem diameters shrank in the day-time and returned to their original size at the night-time. However, with the decrease in availability of soil water, previously contracted stem could not return fully to its original size at nights. When soil water content decreased to 50–60% FC, the daily growth value of stem diameter was negative. Similar results to this one were also observed with fruit trees. These results indicated that SDV was sensitive to changes in plant water status.

There was an apparent diurnal variation in stem diameter due to environmental factors such as solar radiation (Rs) and air vapor pressure deficit (VPD). Three distinct phases were noted within a typical daily SDV cycle on summer days, as reported by previous studies on SDV in trees [34–36]. Fig 1 shows these three phases, namely the shrinkage phase, defined as the period during which the stem diameter decreased, usually from 06:00 to 08:00 h in the morning, increases in incoming solar radiation induced increases in transpiration, as a consequence, leaf water potential and cells turgor decreased, which, in turn, was translated into a shrinkage in stem diameter reaching gradually a daily minimum stem diameter (MNSD) till 14:00–16:00 h; the recovery phase, defined as the portion of the cycle during which the stem diameter started to swell with decreases in solar radiation and VPD until about 06:00 h next morning it reached the value recorded at that morning maximum; and the increment phase, defined as the period during which the stem diameter continued increasing, until it reached a daily maximum stem diameter value (MXSD). Subsequently, the shrinkage phase of the next diurnal cycle begins. However, typically when the plant was under severe water deficit, the stem didn’t undergo an increment phase, and daily growth (DG) values were even negative.

### Effects of stem growth patterns on SDV-derived indices as a water-deficit indicator

**Fig 2:**
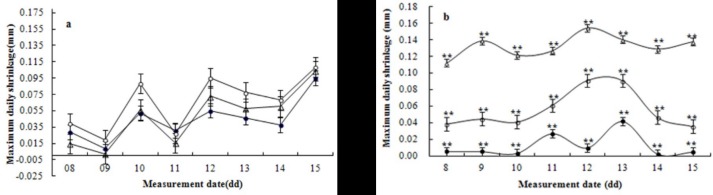
Dynamics of maximum daily shrinkage (MDS) in stem diameter under different growth pattern. Dynamics of MDS during rapid vegetative growth stage of tomato plant from April 8 to 15, 2012 (a) and dynamics of MDS during harvesting stage of tomato plant from June 8 to 15, 2012 (b) in different water treatments (open triangles △: 50–60%FC; open circles ○: 60–70% FC; closed circles ●: 70–80%FC). Each point is the mean of three measurements. Asterisks **indicate statistically significant differences between treatments by LSD_0.01_. Vertical bars correspond to the standard error of observations.

The dynamics of the MDS during flower-fruit stage (rapid vegetative growth phase) under 3 water treatments are shown in Fig 2A. There were no consistent statistically significant differences (P>0.05, LSD) in the MDS values. There were large fluctuations between plants irrigated with different amounts of water; at times the MDS in the well-watered plants was greater than that in the water deficit plants and at other times the reverse was observed. It is uncertain what caused the inconsistencies in the MDS during rapid vegetative growth. It is likely that natural stem diameter growth played a role, i.e., higher growth rates could mask water-related differences in the MDS between plants watered with different amounts of water. It appears that the MDS cannot be used as an indicator of plant water status during flower-fruit stage.

The dynamics of the MDS in stem diameter for 9 successive days at harvesting (i.e., slow vegetative growth stage) of tomato under different water treatments in the greenhouse are shown in Fig 2B. Consistent and statistically significant differences (P<0.01) in the MDS with some fluctuations between plants watered with different amounts were observed in slow-growing mature plants. In the 50–60% FC (moderately deficit watered) and 60–70% FC (slightly deficit watered) treatments, the MDS values remained relatively constant at a range of 0.11–0.15 mm and 0.04–0.09 mm, respectively, with corresponding average values of 6.75 and 3.25 times greater than the 70–80% FC (well-watered plants) treatment. At this time, the MDS values in the 70–80% FC plants were mainly 0.00–0.04 mm. Regression analysis revealed that the MDS was closely related to the SWC (R^2^ of 0.9333; p<0.01), i.e., the MDS in stem diameter increased with a reduction in the SWC. These results suggest that the MDS can be used as an indicator of plant water status during harvest stage (slow vegetative growth stage) of tomato plants.

**Fig 3:**
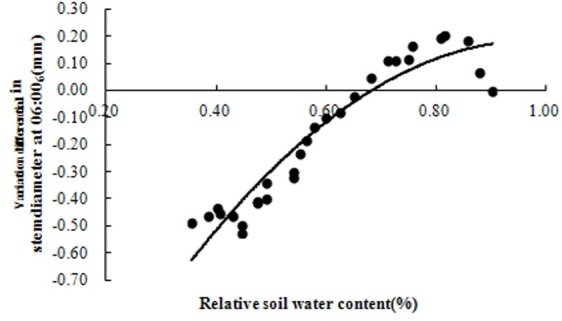
Relationship between variation differential in stem diameter at 06:00 (SD_6_) and relative soil water content (SWC) during rapid growth stage of tomato plant in greenhouse. Correlation equation is y = -2.0721x^2^+4.0684x-1.8121; determination coefficient (R^2^) = 0.9091. Data were collected from April 8 to May 6.

The differences in the daily variation in the stem diameter measured at 06:00 am (SD_6_) (i.e., the difference between the stem diameter value at 06:00 am and the initial reading of the sensor) between the water treatments was constant and statistically significant (p<0.01). Regression analysis indicated a strong quadratic relationship with a determination coefficient (R^2^) of 0.9091 (p<0.01) between the SD_6_ and relative soil water content (SWC) in stem diameter during rapid growth stages (Fig 3). Therefore, the SD_6_ can be used as a sensitive indicator of plant water status in tomato during rapid stem diameter growth. The SD_6_ effectively reflected the natural stem diameter increase and its recovery ability after shrinkage under different water conditions. The SD_6_ was barely affected by climate factors such as solar radiation (R_s_), vapor pressure deficit (VPD) and relative humidity (RH), but was affected by air temperature (T) (Table 2).

**Table 2:** Correlation between SD_6_ at rapidly growing stage and meteorological factors in greenhouse.

Factors	Rs (w.m^-2^)	T (℃)	RH (%)	VPD (KPa)	SD_6_ (mm)	SWC (%)
SD_6_ (mm)	-0.3500	-0.50**	0.2300	-0.3000	1.0000	0.94**

* indicate statistically significant correlation at p<0.05

** indicate statistically significant correlation at p<0.01.

R, solar radiation; T, air temperature; RH, relative humidity; VPD, vapor pressure deficit; SD_6_, daily variation differential in stem diameter at 06:00am; SWC, soil water content.

### Relationships between SDV-derived indices and relative soil water content

Regression analysis was used to establish relationships between the SD_6_ and MDS and SWC (shown in Table 3). The relationships were linear with determination coefficients (R^2^) of 0.9091 and 0.9853 for SD_6_ and SWC, and MDS and SWC, respectively. These relationships were statistically significant (P<0.01), which indicates that SDV-derived indices can sensitively respond to the SWC. Different threshold values for the degree of the soil water deficit, determined by previous research [37], were substituted into the corresponding equations and the SD_6_ and MDS threshold values for detecting plant water status are presented in Table 4. On sunny days, these threshold values can be used as indices for automated irrigation scheduling in tomato plants.

**Table 3:** Relationships between SDV-derived indices and SWC at different growth stages of tomato.

Growth stages	Regression equation	R^2^	n
Rapid vegetative growth stage	SD_6_ = -2.0721 SWC^2^+ 4.0684 SWC—1.8121	0.9091**	29
Slow vegetative growth stage	MDS = -0.5363 SWC +0.7156	0.9853 **	75

** Indicated statistically significant correlation (P< 0.01) by LSD_0.01_ test. SD_6_, daily variation differential in stem diameter at 06:00am; SWC, soil water content; MDS, maximum daily shrinkage.

**Table 4:** Threshold values in SD_6_ at rapid vegetative growth stage and threshold values in MDS at slow vegetative growth stage for diagnosing plant water status of tomato.

		rapid vegetative growth stage	slow vegetative growth stage
Plant water status	SWC (% FC)	SD_6_ (mm)	MDS (mm)
Severe water deficit	35–40%	-0.6420-(-0.5163)	0.5279–0.5011
Moderate water deficit	45–50%	-0.4009-(-0.2959)	0.4743–0.4475
Slight water deficit	55–60%	-0.2013-(-0.1170)	0.4206–0.3938
Well water	75–80%	0.0736–0.1165	0.3134–0.2866

SWC, soil water content; SD_6_, daily variation differential in stem diameter at 06:00am; MDS, maximum daily shrinkage.

### The relationships between the SDV and meteorological variables and its reference equation

Stem shrinkage reflects the redistribution of water reserves due to the modification of water potential gradients and various resistances to water flow within the plant. Because plants are located in the middle of the soil–plant–atmosphere continuum, any measurement of plant water status, if collected during sunlight hours, will depend on the soil water content as well as environmental conditions. Hence, before plant-based water status indicators can be used for irrigation scheduling, a reference value must be obtained in plants under non-limiting soil water conditions.

The results of regression analyses between the MDS in well-watered (70–80% FC, non-limiting soil water conditions) mature plants and environmental variables are presented in Table 5. The MDS was significantly (P<0.01) correlated with Rs, VPD, RH, APR, and AP (r^2^ = 0.3024, 0.8297, 0.7656, 0.3239 and 0.5750, respectively). These results indicated that VPD was the predominant factor affecting the MDS, followed by the RH. A multiple regression equation was derived between the MDS and environmental variables (VPD, RH, Rs, APR and AP), which can be used to establish a reference value for detecting plant water stress based on MDS patterns.

**Table 5:** Relationship between MDS at slow vegetative growth stage of tomato and micrometeorological factors in greenhouse.

Meteorological factors	Regression equations	R^2^	N
Rs (w m^-2^)	MDS = 0.001Rs—0.0075	0.3024**	30
VPD (kpa)	MDS = 0.4543VPD—0.1948	0.8297**	15
RH (%)	MDS = -0.0103RH + 0.5456	0.7656**	15
APR (mmol.m^-2^.s^-1^)	MDS = 0.4483APR—0.0083	0.3239**	30
AP (mb)	MDS = 0.013AP—13.015	0.5750**	30

Rs, solar radiation; VPD, vapor pressure deficit; RH, relative humidity; APR, photosynthetically active radiation; AP, atmospheric pressure; MDS, maximum daily shrinkage

** Indicated statistically significant correlation (P< 0.01) by LSD_0.01_ test.

‘N’ refers to the number of observations used to compute each regression.

$$MDS=\text{-}24.8413+0.8252VPD+0.0141RH+0.0107R_{s}\text{-}4.6728APR+0.0232AP\;(n=30,\;R^{2}=0.9557^{**},\;F=29.49)$$ (1)

**Fig 4:**
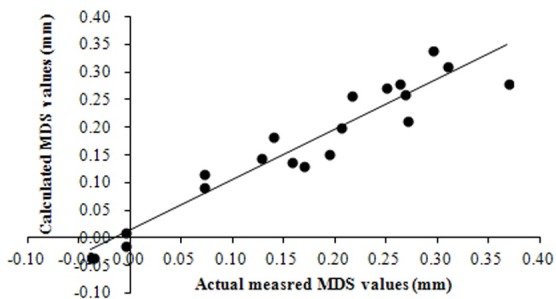
Correlation between MDS values calculated from reference equation and actual measured MDS values. Correlation equation is y = -0.9133x^2^+0.0142x-1.8121; determination coefficient (R^2^) = 0.9133.

The F-test result indicated that the regression equation with a determination coefficient (R^2^) of 0.9557 was statistically significant (P<0.01). The relative error between calculated and measured MDS values was within a range of ± 0.06–5.80%. However, there were no significant differences between calculated and measured MDS values, but there was a statistically significant linear relationship with a determination coefficient (R^2^) of 0.9130 (P<0.01) between MDS values estimated from the reference equation and the actual measured MDS values (Fig 4). Therefore, the regression equation can be used to estimate reference MDS values in tomato plants under non-limiting soil water conditions. When diagnosing plant water status based on SDV-derived indices, a suitable approach is to relate the actual MDS values of a given treatment to the reference MDS values obtained in well-irrigated plants in the same plot. The actual MDS/reference MDS ratio and/or absolute differences between the actual and reference MDS can then be used as an indication of the plant water status to schedule irrigation.

### Relationships between SDV and other plant-based water status indicators

**Fig 5:**
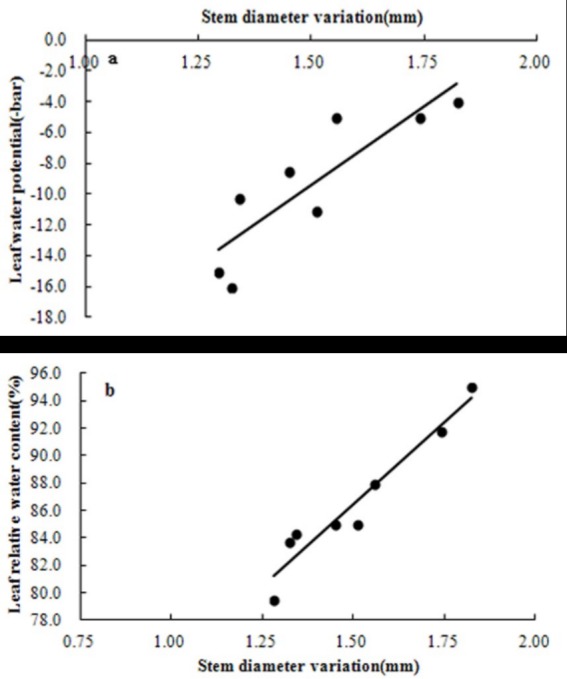
**The relationship between stem diameter variation (SDV) and both leaf water potential (a) and leaf relative water content (b).** Correlation equations are y = -20.339x-39.918 and y = 24.004x+50.489, respectively; determination coefficients (R^2^) are 0.7517 and 0.9312, respectively. Data were from the season in 2011/2012.

The experimental results indicated a linear relationship between the SDV (indicated by the stem diameter value) and the leaf water potential (ψ_L_) using combined data sets from three water treatments (Fig 5A). This linear relationship was statistically significant (P<0.01) and had a determination coefficient (R^2^) of 0.751. The relationship between the SDV and leaf relative water content (LRWC) (Fig 5B) was similar because of the close relationship between the LRWC and ψ_L_; the determination coefficient (R^2^) of 0.751 was statistically significant (P<0.01).

## Discussion

There were apparent differences in the magnitude of the shrinking and swelling of the stem diameter between different soil water conditions, which exhibited various characteristics depending on the phenological stage. During the vegetative growth phase and periods of rapid stem growth, the response of the stem diameter growth to plant water status was more apparent compared with the MDS between water treatments, while there were a few marked differences in daily diameter increase (DI). It is likely that in these plants, the MDS could be affected more by growth than by the level of the water deficit. Under well-watered conditions, stem diameter increased rapidly, thus the DI value was higher, while under water deficit conditions, stem diameter increased slowly, and the DI value was lower or even negative. Similar results were observed in young peach [21], olive [38], lemon [39], and almond [19] trees. In mature plants, and during periods of slow/negligible growth, however, the MDS values in well-watered plants were lower and stem diameter recovered rapidly, whereas the MDS values in water deficit plants were higher, and stem diameters recovered slowly or were not able to recover. The fact that the SDV was characterized by higher DI in rapidly growing young plants compared with a higher MDS in mature plants with little stem growth provided a significant basis for determining suitable SDV-derived indicators to detect plant water status in tomato.

Many researchers across the world have long been searching for useful indicators based on changes in stem diameter and their threshold values in response to plant water status for irrigation scheduling. Stato and Hasegawa [40] demonstrated that the relationship between the relative ratio of stem diameter (RSD) and soil water content (SWC) was significant in greenhouse muskmelon and that an RSD lower than a threshold value could be used as an indicator to start irrigation; however, it is difficult to determine the critical value of the indicator due to changes in the RSD with time over a period of one day. Lee Byun-woo and Shin [41] utilized the daily diameter increase (DI) (i.e., the difference between the stem diameter measured at 6:00 am on two successive days) as the indicator of tomato irrigation and determined DI = 0 as a threshold value for starting irrigation. The DI is closely associated with growth stages. During vegetative growth periods, the stem diameter increases rapidly, resulting in significantly different DI values that correspond to different water conditions. It may be feasible to use the DI as an indicator for starting irrigation. During reproductive growth periods, the stem diameter increases slowly or may cease completely, resulting in similar DI values under different water conditions. As a result, it is not feasible to use the DI as the indicator for starting irrigation during reproductive growth stages. Gallardo et al. [42] found that the ratio of the maximum daily shrinkage (MDS) and air vapor pressure deficit (VPD) maintained relative stability under periods without water stress, and that the MDS/VPD ratio increased significantly under water stress. Therefore, it seems to be more reasonable to utilize the MDS/VPD ratio as the indicator, which is capable of quantifying the effects of VPD on changes in stem diameter. However, Gallardo et al. [42] did not take the correlation between changes in stem diameter and growth stages into account, which resulted in an unreliable diagnosis of plant water status. Based on the studies mentioned above, this paper analyzed and proved that daily variation in stem diameter measured at 06:00 am (SD_6_) produced a quadratic curve relationship that closely approximated the relative soil water content during rapid vegetative growth stages of tomato, whereas during slow vegetative growth stages, the MDS was significantly related to the relative soil water content. As a result, there is more practical significance to select various SDV-derived indices as key indicators for irrigation scheduling according to different stem diameter growth patterns.

Signal intensity (i.e., the actual MDS value / reference MDS value ratio) proposed by Goldhamer and Fereres [21] to assess and compare the sensitivity of indicators to determine plant water deficit has been used extensively in tree species [35, 43, 44]. However, this approach has limited use in tomato; it was unsuitable when the denominator term was close to zero or negative. In our study, the ratio had consistently higher coefficient of variation (CV) values compared with the absolute differences: the CV values were 62–68% for the ratio, compared with 14–37% for the absolute differences. Therefore, the use of absolute differences between actual and reference MDS values from control plants under non-limiting soil water conditions is proposed as an approach for detecting tomato plant water status. For example, the absolute difference between actual MDS values from the 60–70% FC (slightly deficit watered) plants and reference MDS values from the 70–80% FC (well-watered) plants was within a range of 0.03–0.08 mm (Fig 2B), which indicated that there was a slight water deficit in the tomato plants, and the absolute differences between the actual MDS values from the 50–60% FC (moderate deficit watered) plants and reference MDS values from the 70–80% FC plants was within a range of 0.10–0.14 mm, which indicated that there was a moderate water deficit in tomato plants. This method may result in greater management complexity (e.g., different irrigation schedules within the same plots) compared with the use of reference lines, as well as increased investment costs (e.g., a higher number of sensors). However, at a plot scale in the greenhouse, the plants used as the reference can be ‘‘better irrigated” by increasing either the number of drippers or their discharge rate, without the need for different irrigation plots. A superior option would be to develop dynamic simulation models that estimate MDS reference values according to different representative climate year types.

Deficit irrigation strategies and precision irrigation are essential in arid and semi-arid areas where water is scarce. Traditionally, irrigation scheduling decisions are frequently based on the determination of soil moisture content or soil moisture tension. Local measurements of soil water status have, however, the drawback that they do not give direct information about the water needs of a plant [45]. For this reason, the use of plant-based water status indicators has become very popular in recent years for studying plant–water relations and for planning more precise irrigation programmes, because it is recognized that the plant itself is the best indicator of its water status, and the determination of plant-based water stress indicators allows optimum irrigation and thus high efficiency use of the worldwide scarce water resources [46]. Since the plant water status controls many physiological processes and crop productivity, this information can be highly useful in irrigation scheduling [21, 47]. Particularly under deficit irrigation conditions, the continuous control of plant water status is crucial in order to prevent a moderate, potentially beneficial, water stress from becoming too severe and ending in a reduction of the yield [48, 49].

Currently, several methods, such as leaf water potential, stem water potential, vapor diffusion and relative leaf water content, are available for monitoring of plant water status. However, these methods of monitoring plant water status cannot be easily automated and require destruction of plant tissue and all provide intermittent and localized measurements rather than continuous and nondestructive monitoring of plant water status, which may have curtailed the adoption of these techniques for the calculation of irrigation requirements for large areas of farmland. By contrast, the monitoring of plant water status based on SDV-derived indices have the advantages of a simple, reliable, sensitive, nondestructive and continuous data collection and transmission for the whole irrigation season[50, 51, 52], and then can be used as indices for automated irrigation scheduling in tomato plants. This makes it possible to schedule automatic irrigation based on the plant-based water indicators rather than on assessments of climatic factors or soil water content.

There are a variety of factors that may affect SDV derived-indices, such as environmental factors (e.g., soil water availability, Rs, and VPD), and biological factors (e.g., crop species, phenological period, plant age, and plant load), which suggests that stem diameter variations should be considered in the context of the water balance as well as the carbon balance of plants. Moreover, a certain indicator cannot be fit solely for diagnosing plant water status; it is necessary to carefully examine the effects of other related aspects on SDV-derived indicators, especially, dynamic simulation models that estimate MDS reference values as changes in climatic variables should be developed. Multiple-factor reference equation established in this work was just only attempt, which needed to be tested and improved further in the future research and development of irrigation scheduling protocols for tomato.

It is also noteworthy that this experiment was conducted in iron pots. The aim in doing this was to maintain soil water content easily and accurately within prescribed limits of irrigation treatment during the experimental cycle. However, there would have been the probable root growth constraints within the pots. Although the strict control methods were adopted during the experiment, there were some limitations in the current results obtained from pot culture experiments compared to the results obtained from the field experiments.

## Conclusions

Our research with tomato indicated that daily variation in stem diameter measured at 06:00am (SD_6_) was closely related to the relative soil water content (R^2^ = 0.9091, p<0.01) during rapid vegetative growth stage, while during the slow vegetative growth stage, the maximum daily shrinkage (MDS) was closely related to the relative soil water content (R^2^ = 0.9333, p<0.01). The threshold of SD_6_ and MDS for different levels of soil water deficit could be determined by the relationship equations between the both SD_6_ and MDS and the relative soil water content (SWC). These thresholds can be used as an indices of tomato automatic irrigation scheduling.
